# Age and Employee Green Behaviors: A Meta-Analysis

**DOI:** 10.3389/fpsyg.2016.00194

**Published:** 2016-03-02

**Authors:** Brenton M. Wiernik, Stephan Dilchert, Deniz S. Ones

**Affiliations:** ^1^Department of Psychology, University of MinnesotaMinneapolis, MN, USA; ^2^Department of Management, Baruch College, City University of New YorkNew York, NY, USA

**Keywords:** age differences, sustainability, individual environmental performance, employee green behaviors, workplace pro-environmental behaviors, environmental sustainability at work

## Abstract

Recent economic and societal developments have led to an increasing emphasis on organizational environmental performance. At the same time, demographic trends are resulting in increasingly aging labor forces in many industrialized nations. Commonly held stereotypes suggest that older workers are less likely to be environmentally responsible than younger workers. To evaluate the degree to which such age differences are present, we meta-analyzed 132 independent correlations and 336 *d*-values based on 4676 professional workers from 22 samples in 11 countries. Contrary to popular stereotypes, age showed small positive relationships with pro-environmental behaviors, suggesting that older adults engaged in these workplace behaviors slightly more frequently. Relationships with age appeared to be linear for overall, Conserving, Avoiding Harm, and Taking Initiative pro-environmental behaviors, but non-linear trends were observed for Transforming and Influencing Others behaviors.

## Introduction

Interest is growing among corporations and non-profit organizations to reduce the environmental footprints of their operations. Organizational environmental sustainability has been defined as organizations operating in such a way that the present needs of employees, decision makers, and stakeholders are met without compromising the ability of future generations to meet their own needs (Mesmer-Magnus et al., 2012). An increasing number of organizations realize that interventions toward this end need to take into account organizational members to achieve environmental sustainability (Dilchert and Ones, 2012; Ones and Dilchert, 2012b). A recent survey by the Society for Human Resource Management (2011) indicated that nearly two-thirds of the organizations sampled engaged in some kind of environmental sustainability initiative, and about half had a formal policy that addressed workplace sustainability. A systematic investigation of Fortune 500 companies revealed that more than 85% reported environmental sustainability efforts (D'Mello et al., 2011). The majority of these efforts fall into the domains of recycling and reduction of energy use, but pollution prevention and other proactive efforts are also reported (Schmit, 2011). Importantly, involvement from organizational members is essential for most sustainability initiatives (Mesmer-Magnus et al., 2012).

The shift toward a greener economy is creating new occupations and adding new responsibilities to existing occupations to embed environmental sustainability as a core part of job performance (Dierdorff et al., 2013). Research using the O^*^NET taxonomy so far has established more than 60 occupations for which tasks, knowledge, skills, and other characteristics required for successful performance have changed to incorporate environmental aspects (Dierdorff et al., 2013). However, while this shift has created new green jobs and changed the core nature of job performance existing for many jobs, it also now requires employees in all jobs to display behaviors that, while discretionary, contribute to the organization's triple bottom line; environmental performance must be deeply embedded into individual and organizational behavior to reach sustained business success (Anderson and White, 2011; Aguinis and Glavas, 2013). Ones and Dilchert (2009) suggested the label *employee green behaviors* for the “scalable actions and behaviors that employees engage in that are linked with and contribute to or detract from environmental sustainability” (Ones and Dilchert, 2012a, p. 87). Employee green behaviors can be part of any dimension of job performance and can be either required or discretionary, depending on the nature of the job (Campbell and Wiernik, 2015). The burgeoning interest in this important performance domain is based in part on the realization that social, economic, and environmental performance of organizations are interconnected (Elkington, 1998/2002; Jackson, 2012) and that individual performance models need to account for the tripartite composition of organizational performance in order to contribute to organizational sustainability (Ones and Dilchert, 2012b). Thus, it is not surprising that organizations are seeking to understand how employees' behaviors at work affect the natural environment and which personal characteristics lead to good and poor environmental performance at the individual level (investigations so far have included characteristics such as positive affect, Bissing-Olson et al., 2013; personality traits, Kim et al., 2014; job attitudes, Paillé et al., 2013; and personal norms and environmental beliefs, Scherbaum et al., 2008; see Norton et al., 2015 for a review).

At the same time, demographic trends over the last three decades have led to increasingly aging labor forces in many industrialized nations (European Commission (DG ECFIN) and Economic Policy Committee (AWG), 2012). In the U.S., individuals age 45 and older now represent nearly 40% of workers (Bureau of Labor Statistics, 2014). Since 1995, the labor force participation rate has increased for men and women 55 years or older, while holding steady or declining for younger age groups (Mosisa and Hipple, 2006). In addition, an increasing number of adults who reach retirement age decide to stay in the workforce (Pew Research Center, 2009). These well-documented demographic trends have made questions regarding environmentally relevant behaviors of aging workforces increasingly salient.

Common stereotypes reflected in the media and popular press indicate that older individuals are purportedly less environmentally-concerned than younger ones (see Irvine, 2012; Twenge et al., 2012). Older workers are also often characterized as inflexible, unwilling to adopt new habits, and unable to learn new skills (Dennis and Thomas, 2007). Based on these assumptions, organizations and researchers have expressed concern that older workers will be more resistant to changing their work behavior to be more sustainable (e.g., by using tablet technology to reduce paper, using video-conferencing to avoid excessive travel, or generally putting environmental sustainability ahead of personal concerns; cf. The White House Office of the Press, Secretary, 2015). Some authors have also suggested that older workers are more likely to have health problems that prevent many sustainable behaviors (e.g., using the stairs, reducing heating, and cooling use; Afacan, 2015). These age-related stereotypes have led many organizations to express concern that aging workforces will interfere with organizational environmental sustainability goals (Davis-Peccoud, 2013). These concerns have begun to influence management practices in many organizations. For example, beliefs about Millenials' supposedly stronger environmental concern has led environmentally-minded organizations to target young people in recruitment (e.g., Epstein and Howes, 2006; Hasek, 2008; Needleman, 2008; Lancaster and Stillman, 2010; Cachinko Social Recruitment Marketing Solutions, 2011; Ones and Dilchert, 2013; Lui, 2014), practices which disadvantage older workers and place organizations at risk for legal liability (Giang, 2015). In their review of age–job performance relations, Ng and Feldman (2008) observed that similarly negative age-related stereotypes are present for many domains of job performance (e.g., safety performance, interpersonal skills, job dedication, adaptability, computer skills) and influence organizational recruitment, selection, evaluation, and promotion practices. Because negative age stereotypes for environmental sustainability are widespread and have begun to influence human resource management practice, it is important to determine whether these beliefs have any empirical support in reality. The aim of the present paper is to do so by systematically examining age differences in a variety of employee green behaviors.

### Psychological factors suggesting age differences in green behaviors

Besides layperson beliefs about age differences in environmental sustainability, such beliefs are also widespread among environmental sustainability researchers. For example, many environmental psychologists have argued that older individuals are more deeply invested in a “dominant social paradigm” which emphasizes personal concerns and economic growth over environmental well-being, making them less likely to perform pro-environmental behaviors (Dunlap and van Liere, 1978; but cf. Otto and Kaiser, 2014, who argued that repeated exposure to environmental crises over their lifespans may lead to higher levels of environmental awareness among older individuals). Gerontological researchers have also suggested that older individuals' supposed unwillingness to change habits is a key barrier to pro-environmental behavior in aging populations (Pillemer et al., 2011). Many studies have found environmental concern to be higher among younger individuals (see Wiernik et al., 2013, for a meta-analysis), suggesting that older individuals may see less need for environmentally-responsible actions.

Age differences in other psychological characteristics might also contribute to perceived or real differences in employee green behaviors. For example, the personality traits sociability and openness tend to decrease with age (Roberts et al., 2006), and younger workers hold stronger values for adaptability and social relationships (Yeatts et al., 2000; Smola and Sutton, 2002). Older workers are also less willing to learn to use new technologies (Czaja et al., 2006) and tend to prefer stability (Henry, 2000), often to the degree that they will change only when under social pressure or when there are clear benefits to the change (Morris and Venkatesh, 2000). These factors suggest that older workers may be less likely to perform employee green behaviors, especially if those behaviors involve changing habits, using innovative technologies, or interacting with coworkers.

However, older employees also hold stronger values for properly completing work, frugality, and responsibility (Morris and Venkatesh, 2000; Smola and Sutton, 2002), and the personality traits conscientiousness and agreeableness tend to increase with age (Roberts et al., 2006). These characteristics are at the core of many pro-environmental behaviors, such as reducing use, avoiding waste, and proper waste disposal, so age-related differences in these traits suggest that older workers may perform *more* of these behaviors. Thus, while older workers may be less willing to change their habits to benefit environmental sustainability, they may also have stronger natural tendencies to perform resource conservation behaviors with positive environmental impact.

### Research suggesting absence of age differences in green behaviors

While the psychological differences cited above suggest that different categories of employee green behaviors may vary systematically with age, other research suggests that substantial age differences are unlikely. In large scale meta-analyses of 10 dimensions of job performance, Ng and Feldman (2008) observed negligible to weak age relations with core task performance, creativity, training performance, contextual performance, safety performance, and counterproductive behaviors. The only performance dimensions with more substantial age relations were contextual performance directed at tasks and withdrawal behaviors, both of which favored older workers. Similarly, Ng and Feldman's (2012) meta-analysis found that age relations with work attitudes, training participation, proactivity, interpersonal performance, and even support for organizational change efforts were also negligible. The absence of substantial age differences for other domains of work behaviors and attitudes suggests that large differences for employee green behaviors may be unlikely.

A recent meta-analytic investigation of environmental behaviors in *non-work* settings also suggests that age differences in employee green behaviors are likely to be small (Wiernik et al., 2013). Wiernik and colleagues found that relations between age and most environmental behaviors were negligibly small. In fact, *older* individuals were somewhat more likely to engage in behaviors that avoided environmental harm, conserved resources, or involved engaging with the natural world in their personal lives. Based on these results, we expect that age differences in environmental behaviors in work settings will be similarly small. However, there are important differences between pro-environmental behavior in personal life and employee green behaviors (Ones and Dilchert, 2013). Employee behavior in the workplace is typically both more observable and more constrained by organizational requirements and social norms. Individuals also perform different social roles at home vs. at work (Super, 1980). The distinctions between these two contexts suggest that the nomological network of these behaviors in occupational settings could differ notably compared to when they are investigated in non-work settings (Ones and Dilchert, 2012b). With regard to age, for example, organizational rules may require all employees to follow certain waste disposal procedures, attenuating any differences between younger and older employees. Older employees may also have more experience and political resources in organizations; they may be the only employees with sufficient power to implement sustainability initiatives or adopt innovations, leading to a *positive* relation between these behaviors and age. Because of the situational differences between work and non-work settings, there is a need to evaluate whether different categories of employee green behaviors systematically vary with age in the workplace context. Moreover, the implications of age-group differences might be more immediately relevant in organizational settings, potentially necessitating adjustments to human resources interventions such as recruiting, selection, or training in relation to organizational sustainability goals.

### The present study

The present study is a systematic, large-scale investigation of the relations between age and employee green behaviors. Research establishing if and how age groups differ in their environmental performance is crucial in guiding organizations to create and implement initiatives which are effective in bringing about positive environmental change. If older and younger individuals really differ in the frequency and kinds of pro-environmental behaviors they engage in at work, there may be implications for how organizations adapt environmental initiatives—for example through education, socialization, training, job redesign—to meet the needs of specific groups and increasingly age-diverse workforces in general. Such implications are routinely investigated by applied psychologists for many domains of work behavior (e.g., Ng and Feldman, 2008). In this paper, we present the first investigation of age differences in a broad set of employee green behaviors^1^. In doing so, we examine age differences in overall green behaviors as well as specific subdomains. Furthermore, we conduct this investigation in 22 independent samples from 11 countries, in an effort to assess the generalizability of our findings.

For this study, we adopted the conceptualization of employee green behaviors described by Ones and Dilchert (2012a). These authors conducted a large-scale critical incidents study to catalog the full range of environmentally-relevant employee behaviors. Using the results of this study, the authors developed a content-based taxonomy that consists of hierarchically-organized behavioral categories that are successively more homogeneous in their content. The taxonomy consists of 16 specific homogenous subclusters of green behaviors organized into five broad categories—Conserving, Avoiding Harm, Transforming^2^, Influencing Others, and Taking Initiative. The categories are distinguished in terms of their behavioral content (what employees actually do) and achieve conceptual coherence on the basis of their functional core (i.e., what purpose they serve) and psychological underpinnings (individual tendencies and values that motivate the behavior). Descriptions of these five categories, their behavioral subclusters, and example behaviors are provided in Table 1. We adopted the Ones and Dilchert (2012a) taxonomy as an organizing framework for the present study because of its comprehensive, conceptual breadth, and relative parsimony.

**Table 1:** **Descriptions of employee green behavior categories and potential relations with age**.

**Subdomain**	**Definition**	**Behavioral subclusters**	**Behavioral examples**	**Factors potentially influencing age relations**
Conserving	Behaviors aimed at avoiding wastefulness and preserving resources	Reducing use	Turning off lights when not needed; leaving machinery running when idle	*Positive age relations* (+) conscientiousness (+) values for frugality/thrift (+) values for responsibility (+) values for properly completed work *Negative age relations* (−) environmental attitudes (−) environmental concern
		Reusing	Reusing disposable plastic products; relying on single-use products	
		Repurposing	Diverting used cooking oil to make biodiesel; discarding surplus material that could have been used elsewhere	
		Recycling	Recycling cans, bottles, and paper; failing to separate recyclables from trash	
Avoiding harm	Behaviors involving avoidance and inhibition of negative environmental behaviors	Preventing pollution	Treating hazardous waste properly; contaminating soil by dumping toxins	*Positive age relations* (+) conscientiousness (+) values for responsibility (+) values for properly completed work *Negative age relations* (−) environmental attitudes (−) environmental concern
		Monitoring impact	Tracking emissions from operations; failing to clean up after an accident	
		Strengthening ecosystems	Planting trees around work facilities; clearcutting unnecessarily	
Transforming	Behaviors aimed at enhancing the environmental sustainability of work products and processes	Choosing responsible alternatives	Purchasing durable equipment or supplies; using materials from unsustainable sources	*Positive age relations* (+) organizational power *Negative age relations* (−) openness (−) values for adaptability (−) technology attitudes (+) values for stability (−) environmental attitudes (−) environmental concern
		Changing how work is done	Optimizing shipping program to reduce air shipments; knowingly relying on a work process that is energy inefficient	
		Creating sustainable products and processes	Designing a new product to substitute for an environmentally unfriendly one; ignoring environmental impact when designing a new manufacturing process	
		Embracing innovation for sustainability	Choosing virtual meetings instead of travel; insisting on computer printouts when paperless options are available	
Influencing others	Behaviors aimed at spreading sustainability behaviors to other individuals	Educating and training for sustainability	Training employees on recycling procedures; removing environmental content from employee socialization programs	*Positive age relations* (+) agreeableness (+) organizational power *Negative* (−) sociability (−) values for social relationships (−) environmental attitudes (−) environmental concern
		Encouraging and supporting	Encouraging carpooling and helping to coordinate it; asking coworkers to dress warmly instead of using space heaters	
Taking initiative	Behaviors which involve pro-actively initiating new behaviors or making personal sacrifices for sustainability	Initiating programs and policies	Instituting an energy reduction policy; ending an environmental program for business reasons	*Positive* (+) assertiveness (+) organizational power *Negative age relations* (−) openness (−) environmental attitudes (−) environmental concern
		Lobbying and activism	Arguing for environmental issues on board; lobbying for environmentally harmful policies	
		Putting environmental interests first	Turning down an environmentally unfriendly project; not being willing to compromise comfort to reduce energy use	

*Definitions adapted from Ones and Dilchert (2012a). For factors potentially influencing age relations, (+) indicates that prior research shows the factor is higher among older employee and (−) indicates that the factor is higher among younger employees*.

Table 1 also describes psychological factors that may contribute to age differences in each of these employee green behavior categories. For example, behaviors in the Conserving categories share a functional core of thrift and responsibility, so age-related increases in conscientiousness (Roberts et al., 2006) suggest that this category may be positively related to age. Conversely, behaviors in the Transforming category require a degree of adaptability and openness to change, so age-related preferences for workplace stability (Smola and Sutton, 2002) and declines in openness (Roberts et al., 2006) suggest that these behaviors may be negatively related to age. In this study, we examine whether any of these psychological factors manifest as age differences in employee green behaviors and assess whether widely-held age-related environmental sustainability stereotypes have any basis in reality. Our study is intended to guide both researchers and human resources practitioners by empirically establishing the potential relevance of age for employees' environmental performance at work.

## Methods

### Samples and procedure

This study is based on data collected as part of a centrally coordinated, international, multi-organization benchmarking study conducted for a large multinational organization. Data were collected from 11 different countries and at two points in time (wave 1: 2010; wave 2: 2011). The same procedure was employed to recruit one sample from each country in each year, resulting in 22 independent samples, two from each of the following countries: Brazil, China, Germany, Japan, Mexico, Poland, the Russian Federation, Singapore, Switzerland, the United Kingdom, and the United States. The countries sampled come from 6 of the 10 GLOBE regions (Anglo, Confucian, Eastern European, Germanic, Latin American, Latin European; see House et al., 2004) and represent about 35% of the world's population and 60% of the world's economic activity in terms of gross domestic product. Although these countries were selected based on suitability for the benchmarking study, they provide a strong representation of the industrialized world.

Participants within each country were recruited through a professional survey research firm. In total, 4676 employed adults were surveyed; sample sizes ranged from 202 to 224 across the 22 samples. Participants worked in a broad selection of organizations within each country (i.e., sampling was not limited to a single company nor limited to “green” companies, industries, or jobs). They were carefully stratified to be demographically representative of the respective country's professional workforce. Professionals represented the population of interest for the benchmarking study, and accordingly a large majority of participants classified themselves as mid-level management (39.4%), upper-management (26.1%), or top-management (20.4%). The focus on professional workers meant that we could assess a wide range of *discretionary* employee green behaviors from a variety of areas, including those which employees in less complex jobs often do not have opportunity to engage in. These included behaviors that fall into strategic or policy domains or are aimed to encourage pro-environmental behaviors in others (see below).

Overall, participants were employed in more than 23 industries (the survey used a 23-category industry scale but allowed participants to also indicate “other”). Appendix A in Supplementary Material presents the age distributions of the 22 samples. Each sample represents the full range of ages present in the country's professional working population. Deviations from national populations' age medians (obtained from Central Intelligence Agency, 2008) can be attributed to the fact that we studied professional workers, rather than the general population or overall labor force.

### Measures

#### Age

Employee age was measured in years using a continuous scale, allowing for the computation of correlations, as well as age group mean differences once the age variable was polytomized.

#### Employee green behaviors

Survey items were chosen from a larger, pre-calibrated item pool to assess each of the five broad content categories of employee green behaviors described by Ones and Dilchert (2012a) with the goal of picking items that are widely applicable to professional workforces. The original English survey items were professionally translated into the following languages: Mandarin (simplified and traditional), German, Japanese, Portuguese, Spanish (Europe and Latin America), Polish, Russian, and French (as an option in the French-speaking part of Switzerland). Back translation and cultural/linguistic review (carried out by a professional survey translation provider) were used to ensure that the different language versions appropriately reflected the intent of the original survey. Measurement and structural equivalence of the different language versions of the surveys were established (see below for analytic details).

While survey content and structure were equivalent across both years of data collection, format and item number varied slightly. In wave 1, the survey consisted of a 15 item checklist presenting examples of positive environmental work behaviors. For each behavior, respondents indicated whether they had engaged in it on the job in the last 12 months. The 15 items assessed all five subdomains of environmental behavior: Conserving (five items, e.g., “found new uses for discarded or surplus items”), Avoiding Harm (1 item, “disposed of waste properly”), Transforming (five items, e.g., “used innovations to reduce environmental impact”), Influencing Others (two items, e.g., “persuaded others to use environmentally responsible products”), and Taking Initiative (two items, e.g., “behaved in environmentally responsible way even when it was inconvenient”). Originally, several additional items were proposed to assess each of the subdomains. However, due to a variety of organizational constraints, the final survey contained different numbers of items for the five subdomains. Because we were concerned about measurement reliability, we worked with the survey organization to expand the survey to 25 items in wave 2 of the data collection. In this survey, Conserving was measured with six items, Avoiding Harm with four items, Transforming with 10 items, Influencing Others with two items, and Taking Initiative with three items. Additionally, the response format was changed to a 5-point scale measuring the frequency with which employees engaged in the respective behaviors on the job (ranging from “never” to “frequently”).

Items for each subdomain of environmental behavior were summed to obtain a measure of environmental performance in that subdomain. Items were also summed across domains to obtain an employee environmental performance composite, which was used as an indicator of overall environmental sustainability at work^3^. The sustainability composite showed good internal consistency reliability across samples; Cronbach's alpha estimates, which were used to correct observed correlations for attenuation (see below), ranged from 0.71 to 0.83 (*M* = 0.78) for wave 1 samples and from 0.92 to 0.97 (*M* = 0.96) for wave 2 samples.

Factor analysis was used to explore the congruence of the dimensionality of the sustainability composite across samples. Relationships among the subscales of employee green behaviors were uniformly moderate; the range of correlations across 22 samples was 0.48 to 0.59 (*M* = 0.54, *SD* = 0.03). We conducted a factor analysis within each of the 22 samples to examine dimensionality for each of the employee green behavior subscales as well as existence of a latent general factor of employee green behaviors that spans the five subdomains. Such a general factor was found and accounted for an average of 63.6% of the variance in subscale scores across the samples (*SD* = 2.6%, range = 58.3–66.4%). Factor loadings of each of the subscales on the general factor were uniformly moderate across samples (*M* = 0.48, *SD* = 0.05, range = 0.36–0.59). Thus, although each subscale loads on the general latent construct, there is subdomain-specific variance associated with each.

We also assessed measurement invariance by examining the consistency of the factor analytic results across the 11 countries. To this end, we computed congruence coefficients that indicate the degree of similarity between the factor loadings obtained in each country with those obtained in the U.S. sample. Such congruence coefficients can range from −1.00 (maximum inverse similarity) to +1.00 (maximum similarity; see Chan et al., 1999). Typically, coefficients above 0.90 are interpreted as indicating acceptable congruence (Mulaik, 1971; McCrae et al., 1996; Lorenzo-Seva and ten Berge, 2006). The congruence coefficients obtained for the factorial solutions ranged from 0.9591 to 0.9997 (*M* = 0.9891, *SD* = 0.0128), indicating a high degree of similarity between the factor structure of the measure across countries.^4^ In sum, these results suggest that meaningful measurement of overall employee green behavior and its subdomains can be comparatively made across samples. We thus proceeded to meta-analytically combine estimates of the age-employee green behavior relationship across the different country samples.

### Analyses

We analyzed age-employee green behavior relations for the 22 samples using psychometric meta-analysis (Schmidt and Hunter, 2014). In primary research, it can be especially useful to meta-analyze effects from different samples that are similar but come from different contexts. In this case, it is preferable to computing a single effect size for the pooled samples, which would ignore differential reliability across samples as well as the influence of different sample mean levels in both variables (see Ostroff and Harrison, 1999; Waller, 2008, for a detailed discussion). There is also growing interest in using meta-analysis to test generalizability of findings specifically from cross-cultural studies, such as the present investigation^5^. Ones et al. (2012) have laid out the theoretical basis for and empirical approaches in using meta-analysis to test for cross-cultural generalizability. Our study, which meta-analyzes 132 independent correlations and 336 *d*-values from 22 samples in 11 different countries, is an example of what Ones and colleagues' refer to as an “intercontextual approach.” Such an approach is well-suited when one seeks to determine if a true effect is consistently present across different settings. To the extent that statistical artifacts (sampling and measurement error) account for a majority of the variability in effects observed across samples, the corrected estimates of a relationship can be said to generalize. Generalizability is indicated by the 80% credibility interval around a corrected true correlation (ρ) or true group difference (δ); its lower bound is the credibility value above which 90% of true effects in the distribution lie. In line with meta-analytic convention, if the credibility interval does not include zero, we conclude that the relationship between age and environmental performance generalizes across samples.

Before analyzing age-employee green behavior data, we investigated the nature of the relationship to detect potential non-linear effects. To establish adequate power, data were combined within wave (*N*s = 2316 and 2360, respectively). In both cases, a linear model fit the data best. Thus, we first computed correlations between age and the environmental performance scales in each of the 22 samples. These effect sizes were meta-analytically pooled (weighted by sample size and corrected for attenuation due to unreliability in the criterion measures^6^) to arrive at unbiased estimates of true effects and to test for relationships that generalize across samples. The goal of this analytic approach is not only to estimate mean relationships more accurately, but also to investigate whether relationships differ in magnitude across samples once statistical artifacts have been accounted for.

Next, in order to account for potential age differences that might arise due to abrupt maturational shocks (e.g., having children) or meaningful cohort experiences (which themselves could vary across different countries), we also computed and meta-analyzed standardized mean age-group differences in employee green behaviors across countries. For this purpose, we split each of the 22 country samples into four separate age groups. We chose age groups so that they represented relatively homogeneous maturational periods (e.g., early adulthood, career maintenance, late adulthood) while also constituting large enough sample sizes across countries to ensure adequate statistical power. To this end, individuals ranging in age from 36 to 45, 46 to 55, and 56 to 80 years were compared to the 18–35 years-old reference group in each sample. These groups correspond to standard age categories for actuarial and economic research and practice (Frees et al., 2009). We computed Cohen's *d*-values for each group comparison where individual group *N*s were ≥10 and used each sample's total group standard deviation to reference the difference between the means (individual age groups showed no meaningful difference in variability on the employee green behavior composite; the average absolute difference in variability was 9.4%, with no systematic positive or negative pattern along the age gradient). Cohen's *d*-values on the six criterion scales obtained for the 22 samples were then meta-analyzed (weighted by the inverse of the respective effect's sampling error accounting for unequal group sizes; Schmidt and Hunter, 2014, p. 293), correcting for attenuation due to unreliability using a reliability artifact distribution obtained using each country sample's reliabilities.

## Results

**Figure 1:**
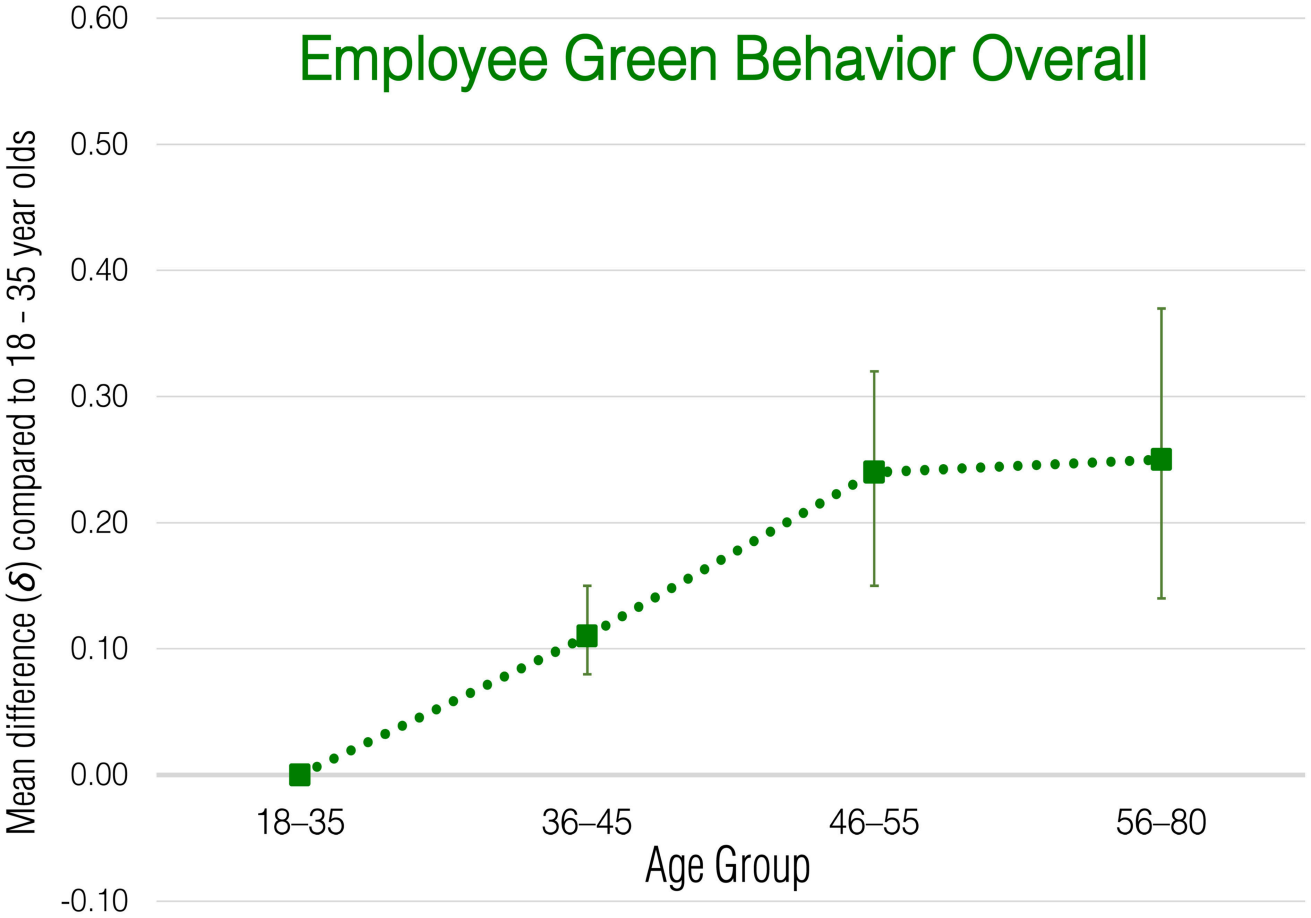
**Meta-analytic age group mean-score differences for overall Employee Green Behaviors**. Error bars indicate 90% confidence intervals around **δ**.

**Figure 2:**
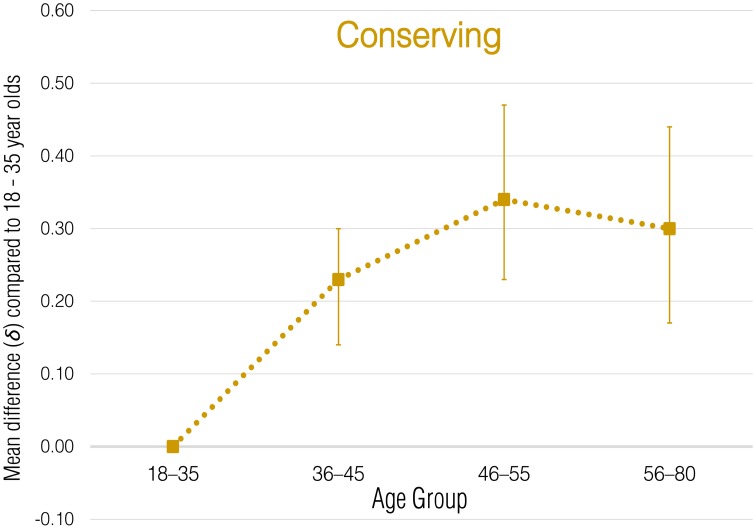
**Meta-analytic age group mean-score differences for Conserving behaviors**. Error bars indicate 90% confidence intervals around **δ**.

**Figure 3:**
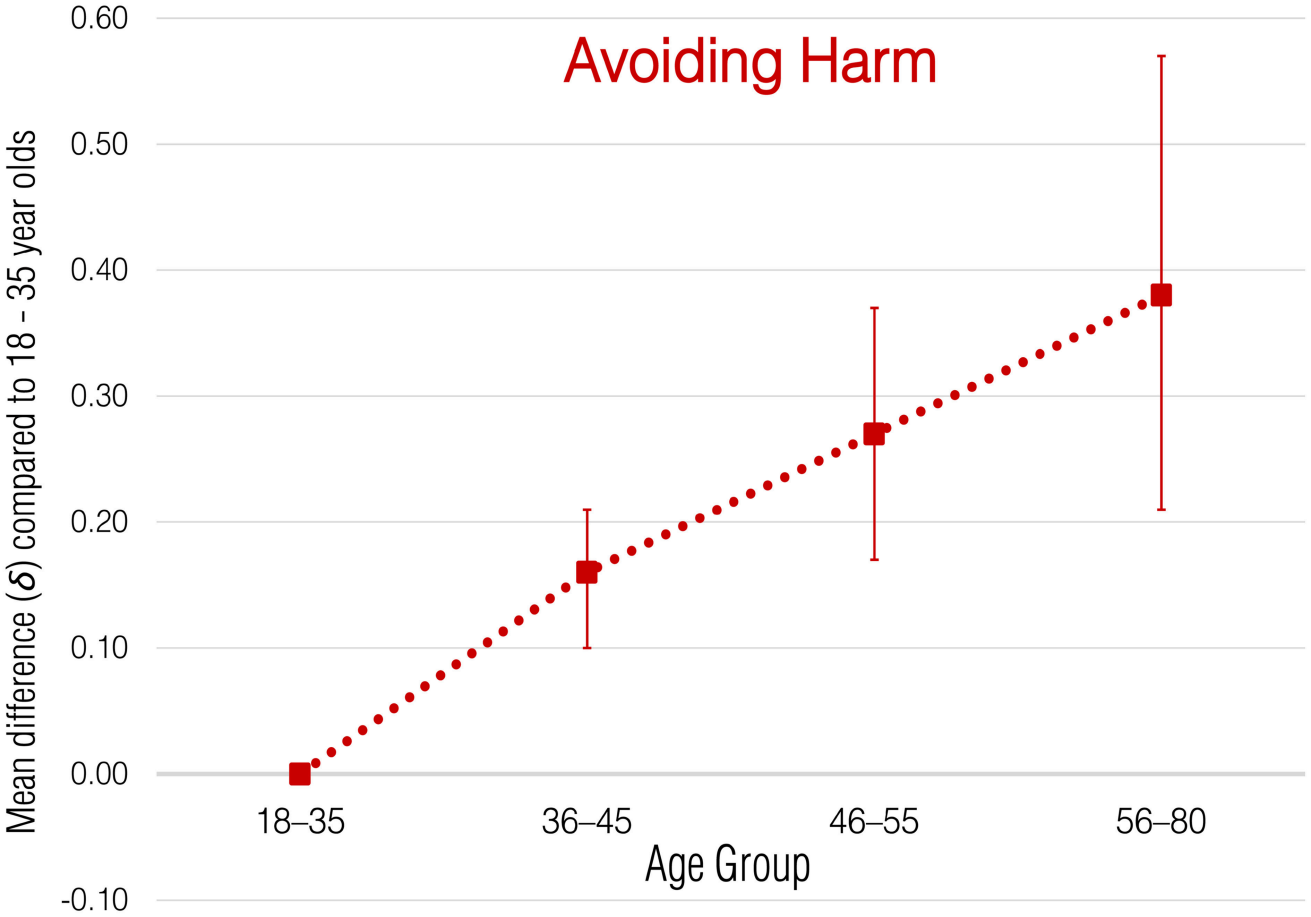
**Meta-analytic age group mean-score differences for Avoiding Harm behaviors**. Error bars indicate 90% confidence intervals around **δ**.

**Figure 4:**
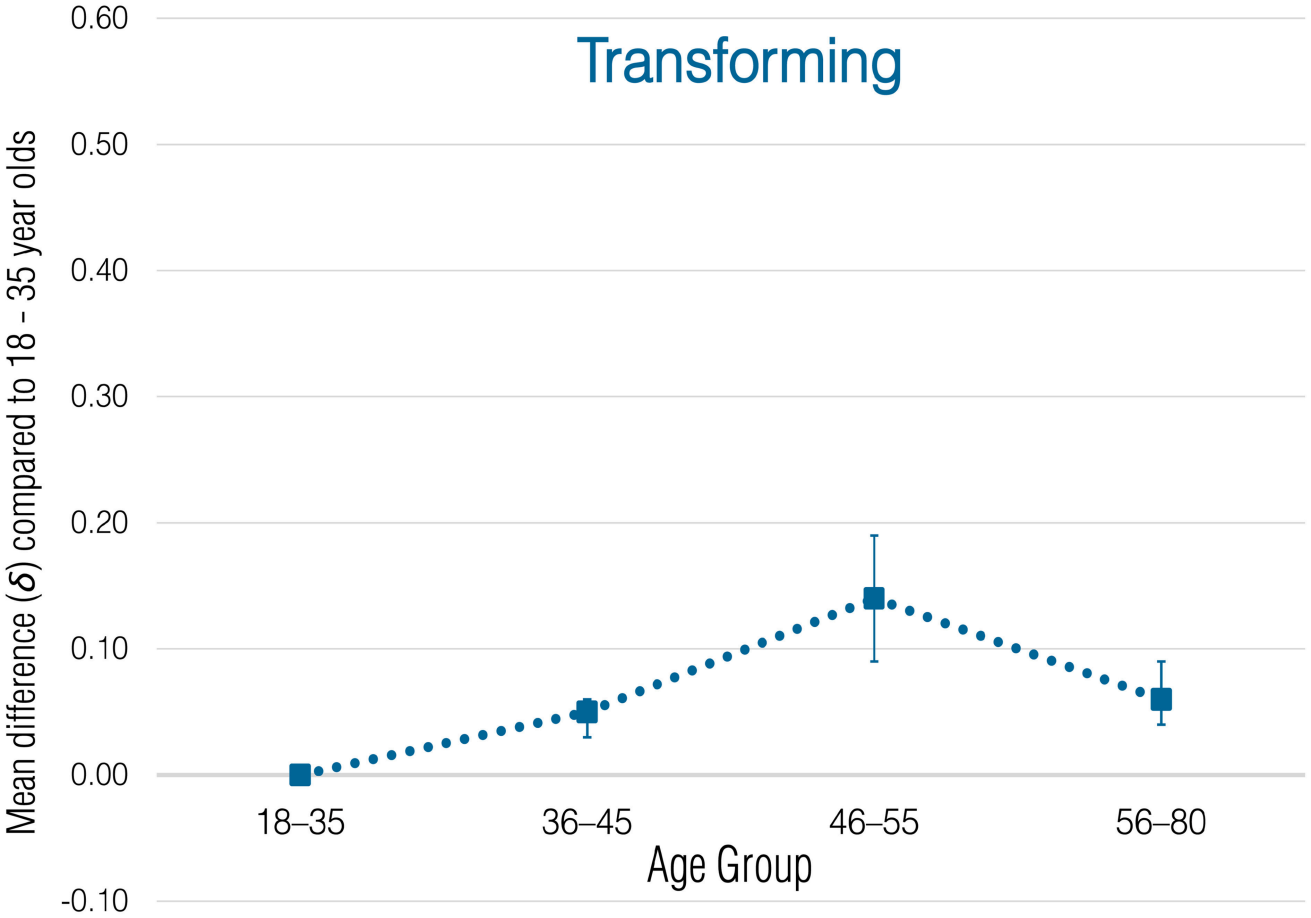
**Meta-analytic age group mean-score differences for Transforming behaviors**. Error bars indicate 90% confidence intervals around **δ**.

**Figure 5:**
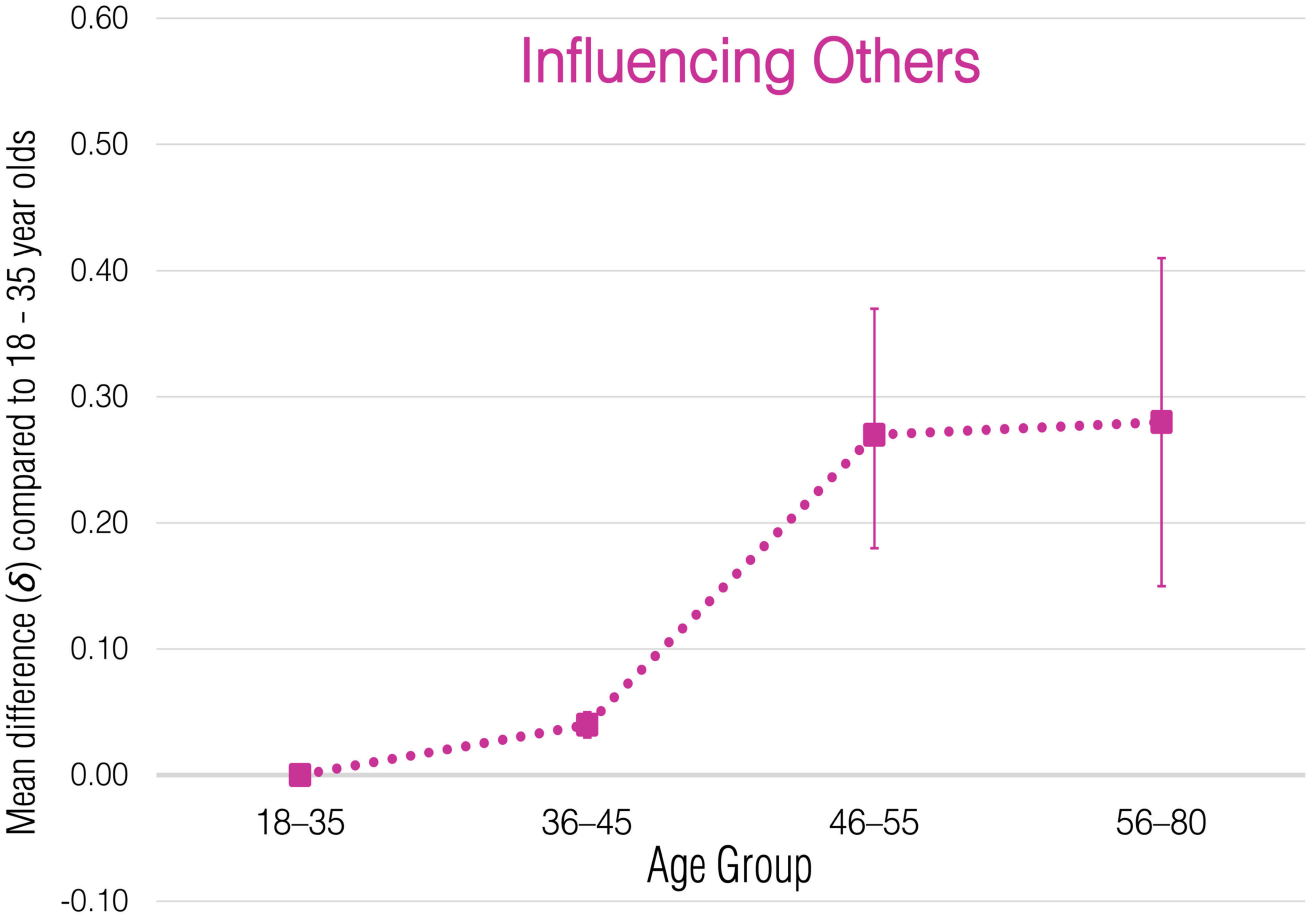
**Meta-analytic age group mean-score differences for Influencing Others behaviors**. Error bars indicate 90% confidence intervals around **δ**.

**Figure 6:**
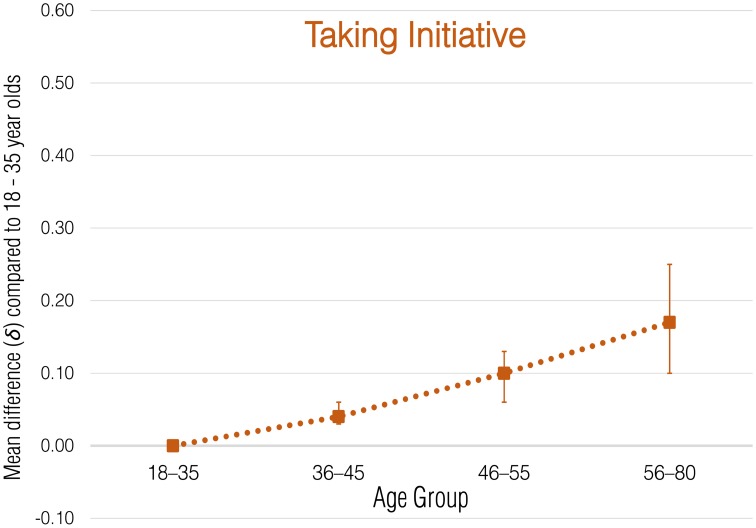
**Meta-analytic age group mean-score differences for Taking Initiative behaviors**. Error bars indicate 90% confidence intervals around **δ**.

Meta-analytic correlational results for overall employee green behavior and each subdomain are presented in Table 2. Meta-analytic results that express these effects in terms of standardized group mean-score differences are presented in Tables 3–8 and illustrated in Figures 1–6.

**Table 2:** **Meta-analytic correlations (ρ) between age and employee green behavior**.

**Environmental performance domain**	***k***	***N***	***mean*$\sqrt{r_{yy}}$**	***r***	***SD*_**r**_**	***SE_r_***	***SD*_res_**	**ρ**	***SD*_ρ_**	**90% CI**	**Credibility interval**
Overall	22	4676	0.93	0.09	0.10	0.02	0.07	0.10	0.08	0.06, 0.14	0.00, 0.20
Conserving	22	4676	0.84	0.10	0.09	0.02	0.05	0.12	0.06	0.09, 0.16	0.04, 0.21
Avoiding Harm^a^	22	4676	0.86	0.10	0.08	0.02	0.05	0.12	0.06	0.08, 0.16	0.05, 0.19
Transforming	22	4676	0.79	0.04	0.09	0.02	0.06	0.05	0.08	0.01, 0.10	−0.04, 0.15
Influencing others	22	4676	0.74	0.09	0.10	0.02	0.07	0.12	0.10	0.07, 0.17	−0.01, 0.25
Taking Initiative	22	4676	0.71	0.04	0.08	0.02	0.05	0.05	0.07	0.01, 0.09	−0.04, 0.14

*k, number of samples included in the meta-analysis; N, total sample size; mean $\sqrt{r_{yy}}$, mean square root of internal consistency reliability estimate ($\sqrt{\alpha }$) across samples; r, sample size weighted mean observed correlation with age; SD_r_, standard deviation of r; SE_r_, sampling error of r; SD_res_, residual standard deviation; ρ, meta-analytic correlation with age, corrected for sampling error, and unreliability in environmental performance measure; SD_ρ_, standard deviation of ρ; CI, 90% two-tailed confidence interval; credibility interval, 80% credibility interval. ^a^In sample 1, Avoiding Harm was measured with a single item. The reported reliability estimate was obtained from the 4-item measure in wave 2, and thus resulted in a conservative correction for attenuation. Results for wave 2 (k = 11) are ρ = 0.14, SD_ρ_ = 0.00*.

**Table 3:** **Meta-analytic age group mean-score differences (δ) for overall employee green behaviors**.

**Age group**	***k***	***N*_younger_**	***N*_older_**	***d***	***SD*_**d**_**	***SE_d_***	***SD*_res_**	**δ**	***SD*_δ_**	**90% CI**	**Credibility interval**
18–35	22	1871		0.00				0.00			
36–45	22	1871	1363	0.10	0.19	0.02	0.07	0.11	0.08	0.08, 0.15	0.01, 0.20
46–55	21	1744	894	0.22	0.28	0.05	0.17	0.24	0.18	0.15, 0.32	0.00, 0.47
56–80	13	742	492	0.23	0.30	0.07	0.18	0.25	0.19	0.14, 0.37	0.00, 0.50

*All age groups are compared to the 18–35 years old baseline. k, number of samples included in the meta-analysis; N, total sample size for the respective age group across samples; d, sample size weighted mean observed group difference; SD_d_, standard deviation of d; SE_d_, sampling error of d; SD_res_, residual standard deviation; δ, sample size weighted mean group difference, corrected for attenuation due to measurement error in the Employee Green Behavior scales; SD_δ_, standard deviation of δ; CI, 90% two-tailed confidence interval; credibility interval, 80% credibility interval. The mean square root of internal consistency reliability estimate ($\sqrt{\alpha }$) across samples was 0.93*.

**Table 4:** **Meta-analytic age group mean-score differences (δ) for conserving behaviors**.

**Age group**	***k***	***N*_younger_**	***N*_older_**	***d***	***SD*_**d**_**	***SE_d_***	***SD*_res_**	**δ**	***SD*_δ_**	**90% CI**	**Credibility interval**
18–35	22	1871		0.00				0.00			
36–45	22	1871	1363	0.19	0.18	0.04	0.04	0.23	0.05	0.14, 0.30	0.17, 0.28
46–55	21	1744	894	0.29	0.23	0.06	0.09	0.34	0.10	0.23, 0.47	0.21, 0.48
56–80	13	742	492	0.25	0.27	0.07	0.13	0.30	0.15	0.17, 0.44	0.10, 0.49

*All age groups are compared to the 18–35 years old baseline. k, number of samples included in the meta-analysis; N, total sample size for the respective age group across samples; $\bar{d}$, sample size weighted mean observed group difference; SD_d_, standard deviation of d; ${SE}_{\bar{d}}$, sampling error of $\bar{d}$; SD_res_, residual standard deviation; δ, sample size weighted mean group difference, corrected for attenuation due to measurement error in the Employee Green Behavior scales; SD_δ_, standard deviation of δ; CI, 90% two-tailed confidence interval; credibility interval, 80% credibility interval. The mean square root of internal consistency reliability estimate ($\sqrt{\alpha }$) across samples was 0.84*.

**Table 5:** **Meta-analytic age group mean-score differences (δ) for avoiding harm behaviors**.

**Age group**	***k***	***N*_younger_**	***N*_older_**	***d***	***SD*_**d**_**	***SE_d_***	***SD*_res_**	**δ**	***SD*_δ_**	**90% CI**	**Credibility interval**
18–35	22	1871		0.00				0.00			
36–45	22	1871	1363	0.14	0.16	0.03	0.00	0.16	0.00	0.10, 0.21	0.16, 0.16
46–55	21	1744	894	0.23	0.24	0.05	0.10	0.27	0.12	0.17, 0.37	0.12, 0.41
56–80	13	742	492	0.33	0.22	0.09	0.00	0.38	0.00	0.21, 0.57	0.38, 0.38

*All age groups are compared to the 18–35 years old baseline. k, number of samples included in the meta-analysis; N, total sample size for the respective age group across samples; $\bar{d}$, sample size weighted mean observed group difference; SD_d_, standard deviation of d; ${SE}_{\bar{d}}$, sampling error of $\bar{d}$; SD_res_, residual standard deviation; δ, sample size weighted mean group difference, corrected for attenuation due to measurement error in the Employee Green Behavior scales; SD_δ_, standard deviation of δ; CI, 90% two-tailed confidence interval; credibility interval, 80% credibility interval. The mean square root of internal consistency reliability estimate ($\sqrt{\alpha }$) across samples was 0.86. Note that in sample 1, Avoiding Harm was measured with a single item—reliability was corrected using the values for the 4-item measure in wave 2 and thus resulted in a conservative correction for attenuation. Results for wave 2 are δ = 0.17, 0.30, 0.35 (SD_δ_ = 0.00 for all analyses)*.

**Table 6:** **Meta-analytic age group mean-score differences (δ) for transforming behaviors**.

**Age group**	***k***	***N*_younger_**	***N*_older_**	***d***	***SD*_**d**_**	***SE_d_***	***SD*_res_**	**δ**	***SD*_δ_**	**90% CI**	**Credibility interval**
18–35	22	1871		0.00				0.00			
36–45	22	1871	1363	0.04	0.17	0.01	0.00	0.05	0.00	0.03, 0.06	0.05, 0.05
46–55	21	1744	894	0.11	0.28	0.02	0.18	0.14	0.23	0.09, 0.19	−0.15, 0.43
56–80	13	742	492	0.05	0.31	0.01	0.20	0.06	0.25	0.04, 0.09	−0.26, 0.39

*All age groups are compared to the 18–35 years old baseline. k, number of samples included in the meta-analysis; N, total sample size for the respective age group across samples; $\bar{d}$, sample size weighted mean observed group difference; SD_d_, standard deviation of d; ${SE}_{\bar{d}}$, sampling error of $\bar{d}$; SD_res_, residual standard deviation; δ, sample size weighted mean group difference, corrected for attenuation due to measurement error in the Employee Green Behavior scales; SD_δ_, standard deviation of δ; CI, 90% two-tailed confidence interval; credibility interval, 80% credibility interval. The mean square root of internal consistency reliability estimate ($\sqrt{\alpha }$) across samples was 0.79*.

**Table 7:** **Meta-analytic age group mean-score differences (δ) for influencing others behaviors**.

**Age group**	***k***	***N*_younger_**	***N*_older_**	***d***	***SD*_**d**_**	***SE_d_***	***SD*_res_**	**δ**	***SD*_δ_**	**90% CI**	**Credibility interval**
18–35	22	1871		0.00				0.00			
36–45	22	1871	1363	0.03	0.19	0.01	0.06	0.04	0.08	0.03, 0.05	−0.06, 0.14
46–55	21	1744	894	0.20	0.26	0.04	0.14	0.27	0.19	0.18, 0.37	0.03, 0.51
56–80	13	742	492	0.21	0.32	0.06	0.21	0.28	0.28	0.15, 0.41	−0.08, 0.64

*All age groups are compared to the 18–35 years old baseline. k, number of samples included in the meta-analysis; N, total sample size for the respective age group across samples; $\bar{d}$, sample size weighted mean observed group difference; SD_d_, standard deviation of d; ${SE}_{\bar{d}}$, sampling error of $\bar{d}$; SD_res_, residual standard deviation; δ, sample size weighted mean group difference, corrected for attenuation due to measurement error in the Employee Green Behavior scales; SD_δ_, standard deviation of δ; CI, 90% two-tailed confidence interval; credibility interval, 80% credibility interval. The mean square root of internal consistency reliability estimate ($\sqrt{\alpha }$) across samples was 0.74*.

**Table 8:** **Meta-analytic age group mean-score differences (δ) for taking initiative behaviors**.

**Age group**	***k***	***N*_younger_**	***N*_older_**	***d***	***SD*_**d**_**	***SE_d_***	***SD*_res_**	**δ**	***SD*_δ_**	**90% CI**	**Credibility interval**
18–35	22	1871		0.00				0.00			
36–45	22	1871	1363	0.03	0.18	0.01	0.03	0.04	0.04	0.03, 0.06	−0.01, 0.09
46–55	21	1744	894	0.07	0.25	0.01	0.14	0.10	0.20	0.06, 0.13	−0.15, 0.35
56–80	13	742	492	0.12	0.22	0.03	0.00	0.17	0.00	0.10, 0.25	0.17, 0.17

*All age groups are compared to the 18–35 years old baseline. k, number of samples included in the meta-analysis; N, total sample size for the respective age group across samples; $\bar{d}$, sample size weighted mean observed group difference; SD_d_, standard deviation of d; ${SE}_{\bar{d}}$, sampling error of $\bar{d}$; SD_res_, residual standard deviation; δ, sample size weighted mean group difference, corrected for attenuation due to measurement error in the Employee Green Behavior scales; SD_δ_, standard deviation of δ; CI, 90% two-tailed confidence interval; credibility interval, 80% credibility interval. The mean square root of internal consistency reliability estimate $\left(\sqrt{\alpha }\right)$ across samples was 0.71*.

### Age and overall environmental performance

Popular stereotypes suggest that younger workers should outperform older workers in environmental performance. However, the corrected meta-analytic correlation between age and the overall employee green behavior composite suggested a weak *positive* relationship with age (ρ = 0.10). The results for age group mean comparisons suggest that the increase in employee green behaviors among older workers is most apparent between the ages of 36 and 55 (δ = 0.11 for the 36–45 group and 0.24 for the 46–55 group); performance of pro-environmental behaviors was similar for the 46–55 and 56–80 age groups. Age relationships with overall green behaviors were somewhat variable across samples, though remained consistently small.

### Age and subdomains of environmental performance

#### Conserving

By far the most commonly observed subdomain of employee green behaviors is Conserving (Ones and Dilchert, 2012a); its functional core relates to frugality and thrift. For this domain, the corrected meta-analytic correlation was ρ = 0.12 with small variability (*SD*_ρ_ = 0.06), indicating that older individuals generally exhibited these behaviors at a higher rate. There was a uniformly positive relationship between age and Conserving behaviors across samples, even though the magnitude of the relationship varied to some degree. The credibility interval around the estimate of ρ did not include zero (0.04, 0.21), indicating that once variation due to sampling error and unreliability was accounted for, the relationship generalizes. Consistent with the results for overall employee green behaviors, age group mean differences in Conserving were most apparent for the 36–45 (δ = 0.23) and 46–55 (δ = 0.34) age groups.

#### Avoiding harm

Conceptually, Avoiding Harm behaviors are similar in nature to avoiding counterproductive work behaviors (e.g., avoiding pollution), as well as to task-based organizational citizenship behaviors and safety performance (e.g., disposing of hazardous waste properly), dimensions of job performance on which older workers slightly outperform younger workers. Thus, we might expect that if any domain of employees' environmental performance were to show age differences, it would be Avoiding Harm. Results showed some support for this expectation. The meta-analytic correlation between age and Avoiding Harm was small and positive (ρ = 0.12; *SD*_ρ_ = 0.06) and generalizable (lower 80% credibility value = 0.05). Results for the group mean difference meta-analyses showed consistent small increases in Avoiding Harm behaviors across all age groups (δ = 0.16, 0.27, and 0.38 for each successive age group).

Note that in the first year of data collection, this aspect of employee green behaviors could only be measured with a single item, so it was not possible to estimate internal consistency reliability. In applying corrections for attenuation due to measurement error in the environmental sustainability measure, only the (much higher) reliability estimates from the second year of data collection (4-item scale) could be used, resulting in underestimates of the true effect in year 1. Considering only the results from the second year of data collection, the meta-analytic correlation is ρ = 0.14 (*SD*_ρ_ = 0.00) and the group mean differences are δ = 0.17, 0.30, and 0.35 for the 36–45, 45–55, and 56–80 age groups, respectively (*SD*_δ_ = 0.00 for all analyses).

#### Transforming

Observed correlations with age varied widely across samples for Transforming (range = −0.21 to 0.26). Most correlations were positive, but negative correlations were observed for some samples (Japan sample 1, Russian Federation sample 1, and United States sample 2, *r*_corrected_ = −0.11, −0.16, and −0.21, respectively). Because of this variability in direction and magnitude across samples, the meta-analytic estimate of the mean effect was very small with comparatively large unexplained variance (ρ = 0.05, *SD*_ρ_ = 0.08). The credibility interval included zero (−0.04, 0.15). Thus, the relation between Transforming and age appears to somewhat variable across settings, ranging from negligible to weakly positive.

An examination of the group mean score differences (see Table 6 and Figure 4) reveals a potential explanation for the negligible relationship between this employee green behavior domain and age. While older workers in the age groups of 36–45 (δ = 0.05) and 46–55 (δ = 0.14) on average scored increasingly higher to a small degree, individuals in the oldest age group showed a decrease back toward the level of the young reference group of 18–35 years-olds (δ = 0.06). This non-linear pattern in age group mean score differences might be founded in the types of behaviors included in this sustainability subdomain, many of which relate to the use of innovative technology. Adapting to new technologies is one dimension of job performance that does show substantial negative relations with employee age (Ng and Feldman, 2008). It appears that Transforming is one category of employee green behavior where significantly older employees (i.e., employees older than 55, which represent a small proportion of employees in most workforces) do show performance declines. However, despite these declines, even the oldest employees perform more of these behaviors than do younger individuals.

#### Influencing others

In terms of individual country correlations, the relationship between age and Influencing Others was the most variable across countries (*r*_corrected_ range = −0.15 to 0.63). Once sampling error was accounted for, the meta-analytic estimate of the mean effect was weakly positive (ρ = 0.12) and considerably less variable across settings (*SD*_ρ_ = 0.10). The 80% credibility interval ranged from a negligible relationship to a moderate positive relationship (−0.01, 0.25). Thus, we can conclude that while the relationship is variable, in most settings, age and Influencing Others are weakly to moderately positively related. Results for the age group mean differences suggest that this relationship results from an abrupt increase in Influencing Others behaviors between the 35–45 (δ = 0.04) and 46–55 (δ = 0.27) age groups. This is consistent with the nature of the behaviors in this domain. As employees enter middle age, they are more likely to move into higher level managerial and leadership positions that afford them the opportunity to encourage and motivate other employees, implement training programs, and provide support for environmental performance of others.

#### Taking initiative

Of all the age-employee environmental performance relationships investigated in this study, the findings for Taking Initiative were the smallest across samples (range = −0.12 to 0.15). The small magnitude of the meta-analytic correlation (ρ = 0.05, *SD*_ρ_ = 0.07) and the wide credibility interval (−0.04, 0.14) indicate that age was not systematically related to Taking Initiative behaviors across samples. Results were similar for the group mean comparisons; each successively older age group showed only a very small increase in Taking Initiative behaviors (δ = 0.04, 0.10, and 0.17).

## Discussion

The present research expanded on previous work on age differences in job performance by investigating the relationship between age and the emerging criterion domain of employee green behaviors. Parallel to other performance domains, popular stereotypes suggest that older workers might lag behind in environmentally relevant job behaviors. We examined age relationships both for overall environmental performance as well as for conceptually and empirically distinct subdomains of employee green behaviors. We conducted an intercontexual meta-analysis of 22 samples from 11 countries (total *N* = 4676). We found that, contrary to popular stereotypes, age showed generally small positive relationships with environmental performance. For some subdomains of environmental performance, and for several age group comparisons, these positive relationships were found to generalize across countries and samples. Older employees appeared to be slightly more likely to engage in conserving behaviors, to expend more effort to avoid environmental harm in the workplace, and to encourage and promote environmental sustainability among other employees. Ones et al. (2012) suggested that intercontextual research designs, such as the one employed in the present study, are useful for examining the cross-cultural generalizability of relations between variables, as such analyses can determine whether observed differences across countries are attributable to statistical artifacts. The narrow width of most credibility intervals and the cultural diversity of the countries sampled suggests that culture is not likely to be a major moderator of age-employee green behavior relations.

The results of our meta-analyses of workplace environmental behavior parallel results found in Wiernik et al.'s (2013) meta-analysis of age differences in environmental sustainability in non-work settings. Wiernik et al. found that age relations with pro-environmental behaviors were small in magnitude and favored older individuals. The present study establishes that relations are similar in direction and magnitude in work contexts. The consistency in results across work and non-work settings indicates that differences between these contexts (e.g., varying levels of autonomy, power, and observability, differing social roles) do not have a moderating impact on age-employee green behavior relations. While these situational factors may exhibit main effects on environmental behavior and moderate the effects of other variables (e.g., the power of social norms to change behavior; Ones and Dilchert, 2012b), individual age remains consistently modestly related to green behavior across settings.

The findings of our meta-analyses within this new performance domain also parallel those of Ng and Feldman (2008), who examined age relationships for major dimensions of job performance, such as core technical performance, creativity, training performance, organizational citizenship behaviors, and counterproductive work behaviors. For the most part, Ng and Feldman also reported small correlations that slightly favored older employees, dispelling commonly held stereotypes that older employees exhibit lower performance compared with their younger counterparts. The same appears to be true for the employee environmental performance domain. Despite commonly held notions about older employees' slow adoption of environmental sustainability efforts, we found no appreciable age differences.

The modest relations observed between age and employee green behaviors should not be interpreted as evidence that the age-related socio-psychological factors discussed in the introduction and in Table 1 are unrelated to employee green behaviors. Rather, the results merely indicate that age is a poor proxy for these variables when studying environmental sustainability. Indeed, several of these variables have been shown to have important impacts on employee green behaviors (e.g., personality traits, Kim et al., 2014; environmental attitudes, Scherbaum et al., 2008). Researchers who are interested in examining the impact of environmental attitudes, personality traits, work values, organizational power, or other factors on employee green behaviors, should measure these focal variables directly, rather than relying on deficient proxy variables such as employee age.

As organizations move toward greater environmental responsibility (see Schmit et al., 2012), employee contributions to organizations' environmental sustainability efforts will be increasingly important. Age stereotypes may lead human resources managers to worry that older employees will hinder their organizations' attainment of environmental sustainability goals. However, the results of this study suggest that these fears are mostly unsubstantiated. Older individuals actually perform some pro-environmental behaviors at work (Conserving, Avoiding Harm, and Influencing Others) at higher rates than younger individuals. It should be noted, however, that while positive trends between age and environmental behaviors were observed, these age differences were small in magnitude. As a result, employee age is likely to have a minor impact on organizational environmental sustainability. These results have implications for interventions to address organizational environmental goals. Human resources practitioners might be concerned that incorporating environmental performance criteria into performance management systems might unfairly disadvantage older workers, but this does not appear to be the case. Perhaps more importantly, organizations seeking to improve their environmental performance should not be concerned that older workers will impair their efforts. Given that age differences in environmental performance are small in magnitude, and actually run counter to commonly held assumptions, preferring younger individuals in employee selection or other staffing decisions would be both unfair and counterproductive with regard to achieving environmental goals. Designing effective human resources interventions will be key to improving employee environmental performance in organizations, and only relevant employee characteristics should be considered in their design.

### Directions for future research

We view the present research as a major step toward understanding age differences in employee environmental performance. We used a comprehensive meta-analytic approach to assess employee green behaviors using multiple samples from several countries. Future research should expand upon our methods and results to improve our understanding of environmental sustainability at work, the aging process in the workplace, and their relationship to one another.

One avenue for future research will be the inclusion of additional countries to further our understanding of the impact employees are able to have on organizational environmental sustainability. Even though the 11 countries sampled in our study represented six different cultural regions, including additional countries and contexts (particularly less industrialized world regions) will enable stronger tests of the cross-cultural generalizability of our findings of negligible age-environmental performance relationships. More and larger samples are also needed to confirm the non-linear relationships between age and Transforming and Influencing Others behaviors. We encourage replications of this research, both in additional countries as well in those already investigated (cf. Carpenter, 2012).

For some subdomains of sustainability (Transforming, Influencing Others, and Taking Initiative), there was a substantial amount of residual variation in age group differences after accounting for sampling error and unreliability (e.g., *SD*_δ_ = 0.23 for Transforming for the 46–55 to 18–35 age group comparison). In *post hoc* analyses, we examined several possible substantive moderators of these group differences, such as cultural characteristics, sample mean age, and base rates of employee green behaviors. None of these moderators showed substantial relations with observed group differences. Indeed, the variation appeared to be due to a small number of extreme outlier values; removing the most extreme value from either end of the distribution reduced the residual variation to negligible amounts for each of these behavioral domains. These extreme values were generally present in only one of the two samples from those countries (e.g., *d*_46–55_ = 0.71 for Influencing Others for Mexico sample 1, but 0.05 for Mexico sample 2). As a result, we conclude that these estimates of *SD*_δ_ for the selected sustainability subdomains are due to second order sampling error (Schmidt and Hunter, 2014, Chapter 9), rather than systematic moderators such as cultural differences or demographic sample composition. This conclusion is bolstered by the observation of comparatively less residual variation for the meta-analyses of correlations (where the respective effects are based on larger sample sizes). Future studies of age-employee green behavior relationships in additional countries will help to clarify whether this observed variability is indeed artifactual.

Second, like most studies that examine age in work settings (e.g., Ng and Feldman, 2008, 2012), our study used a cross-sectional design. While such studies are useful in guiding organizational decision making and theorizing about the antecedents of important workplace behaviors and outcomes, there have been increasing calls for longitudinal research in applied psychology to better understand how workplace processes and individual workplace behaviors unfold and change over the course of individuals' lives (Baltes et al., 2012). As such work develops and increases in popularity, researchers should include environmental performance criteria among the behavioral domains studied. Longitudinal research on relationships between age and environmental performance will be able to disentangle aging and maturation from generational cohort effects (see Footnote 1). Even though the distinction between aging and generational effects is of little consequence for *today's* organizations (i.e., environmental sustainability needs to be addressed in a given workforce at one point in time, and the practical need for interventions is the same regardless of the source of observed age differences), a better understanding of age and developmental effects will be critical for long-term organizational planning, as well as for making environmental policy decisions from a societal perspective. Of course, given the unique generational groups that exist in many cultures, such longitudinal research will have to be conducted within specific cultural settings, which will complicate investigations of the generalizability of results.

Our study is characterized by several major strengths. We examined correlations of age with overall employee green behaviors as well as its subdomains. We also used age group mean score comparisons to detect effects that might have been masked in a strict correlational design. The use of meta-analysis with our multi-country primary samples helped us reach conclusions regarding generalizable effects by correcting for the biasing influence of sampling and measurement error. Direct replications using such large numbers of samples in different cultural contexts are extraordinarily scarce in organizational behavior research (see Spector et al., 2001; Albrecht et al., 2014, for two rare exceptions). In sum, the present study not only provides the first investigation of age differences in this important new performance domain, but does so at a scale that is typically only matched in quantitative summaries spanning several decades of published research.

In sum, despite rampant stereotypes downplaying the willingness and ability of older employees to positively contribute to environmental sustainability, the reality appears to be that such stark age differences in environmental performance do not exist. As industrial, work, and organizational psychologists help design effective strategies and interventions for increasing green behaviors at work, they can do so without being concerned about their differential impact on employees of different ages. By basing practice on empirical reality, rather than on unfounded preconceptions, we can ensure that our efforts succeed in furthering the environmental sustainability of organizations.

## Ethics statement

The research presented in this manuscript has been determined as exempt from Human Research Protection Program (HRPP) and Institutional Review Board (IRB) review at the City University of New York, because it did not involve human subjects as defined by CUNY HRPP policy. The retrospective data analysis did not contain privately identifiable information.

## Author contributions

All authors listed, have made substantial, direct and intellectual contribution to the work, and approved it for publication.

### Conflict of interest statement

The authors declare that the research was conducted in the absence of any commercial or financial relationships that could be construed as a potential conflict of interest. The reviewer DJ and handling Editor declared their recent collaboration, and the handling Editor states that the process nevertheless met the standards of a fair and objective review. The reviewer CW and handling Editor declared their recent collaboration, and the handling Editor states that the process nevertheless met the standards of a fair and objective review.

## Footnotes

^1^When applied psychologists examine age differences in the workplace, they are typically interested in how age relates to relevant work behaviors at one point in time (see, for example, Ng and Feldman, 2008, who meta-analyzed cross-sectional age differences on 10 dimensions of job performance). This is because any observed age differences on relevant criterion variables have important workforce management implications for organizations, regardless of whether they stem from maturation or generational effects, or both. Aging research sometimes employs longitudinal investigations to disentangle these two types of effects. Longitudinal and cross-sectional designs answer different questions and are associated with their own set of deficiencies (cf. Baltes, 1968). In line with existing workplace research on age, our research is focused on age differences in environmental performance at one point in time as well as with the implications of these differences for organizational policies and interventions.

^2^This category was initially termed “Working Sustainably” in the Ones and Dilchert (2009, 2012a) taxonomy, but was renamed to better reflect construct meaning (the behavioral content and subclusters have remained unchanged).

^3^We also formed an overall environmental sustainability composite by weighting items by the inverse of their respective base-rates within each country, to account for potential differential item difficulty (i.e., rarity) across behaviors, which might itself vary across countries. Similarly, we also formed a total sustainability composite by averaging across sustainability subdomain scores (this composite correlated 0.99 with the summed composite). Results for both types of alternative composite mirrored those presented in the present analyses, and thus the more straightforward analyses are presented here.

^4^More detailed results of the factor analyses described in this section are available from the first author.

^5^Apropos of the aging vs. generational effects discussed in Footnote 1, it is important to note that examining generational effects using cross-cultural data is extremely difficult. Each country or region is subjected to unique cultural influences that can lead to the formation of unique generations. Generations that emerge during the same time period in different countries are often subjected to different experiences, and thus cannot be regarded as homogeneous generational units. To illustrate how cross-cultural generations might differ at the same point in time, consider the following examples from countries included in our data collection: The U.S. American “Baby Boomer” generation (1946–1965) grew up during a time of widespread affluence and developed a strong sense of individualism (see Strauss and Howe, 1991). At the same time, the Chinese generation growing up during the “Consolidation Era” (roughly 1950–1965) was subjected to Communist Party policy that placed the communal order above individual and family concerns (Egri and Ralston, 2004). The corresponding generation in Russia is described as cynical and as having goals of exploiting the state's system for their own personal advantage (the “Normal Generation”; Mishler and Rose, 2007), while the German “68er Generation” (born roughly between 1945 and 1960) is often identified with its radical quest to leave behind established structures associated with the war generations of their parents. Whereas generational characteristics across cultural contexts are often dissimilar, age is a variable that has equivalence across cultures. Relationships between generational groups and work behaviors often cannot be meaningfully examined in multinational data, providing yet another reason why this manuscript focuses on overall age-effects (see Footnote 1).

^6^In wave 1, Avoiding Harm was measured with a single-item, so coefficient alpha could not be computed. To correct these correlations for attenuation due to unreliability, we used the reliability estimates obtained from each country for the longer Avoiding Harm measure used in wave 2. This resulted in undercorrection and conservative estimates of age-Conserving relations. We consider the implications of this undercorrection when discussion the results for Avoiding Harm in Section Avoiding Harm.
