# Supplementary material for: Age and Employee Green Behaviors: A Meta-Analysis
Source: Front Psychol. 2016 Mar 2;7:194. doi: 10.3389/fpsyg.2016.00194 (PMC4773609; doi:10.3389/fpsyg.2016.00194)
Supplement: Supplementary file 1 [file DataSheet1.pdf]

Appendix A. Age distributions of 22 samples across 11 countries.

Sample	Sub-sample	*N*		% of sample		% of nat'l pop.	Age range		Sample age *M*		Sample age *SD*		Sample age median		Nat'l age median
		wave 1	wave 2	wave 1	wave 2		wave 1	wave 2	wave 1	wave 2	wave 1	wave 2	wave 1	wave 2	
Across countries	<40	1,279	1,197	55.2	50.7		18-39	18-39	30.9	31.3	5.2	4.9	31.0	32.0	
	≥40	1,037	1,163	44.8	49.3		40-82	40-84	50.2	50.8	7.6	8.6	49.0	49.0	
	Total	2,316	2,360	100.0	100.0		18-82	18-84	39.5	40.9	11.5	12.0	38.0	39.0	
Brazil	<40	166	119	79.4	55.1	56.4	18-39	25-39	28.7	32.3	5.2	4.3	28.0	32.0	
	≥40	43	97	20.6	44.9	43.6	40-67	40-65	47.6	48.6	6.7	6.1	45.0	48.0	
	Total	209	216	100.0	100.0	100.0	18-67	25-65	32.6	39.6	9.5	9.6	30.0	39.0	28.9
China	<40	173	150	82.4	67.6	49.7	21-39	20-39	32.0	32.0	4.4	4.6	32.0	32.0	
	≥40	37	72	17.6	32.4	50.3	40-61	40-60	44.5	46.0	4.8	5.5	43.0	45.0	
	Total	210	222	100.0	100.0	100.0	21-61	20-60	34.2	36.5	6.5	8.2	34.0	36.0	35.2
Germany	<40	65	110	30.4	49.8	34.7	26-39	19-39	34.3	29.7	3.9	5.1	35.0	30.0	
	≥40	149	111	69.6	50.2	65.3	40-68	40-71	50.1	49.3	7.2	8.5	49.0	47.0	
	Total	214	221	100.0	100.0	100.0	26-68	19-71	45.3	39.6	9.7	12.1	45.0	40.0	44.3
Japan	<40	33	81	16.0	38.0	36.3	26-39	23-39	34.3	35.5	3.5	3.5	35.0	37.0	
	≥40	173	132	84.0	62.0	63.7	40-82	40-82	51.8	51.8	8.0	6.5	51.0	51.0	
	Total	206	213	100.0	100.0	100.0	26-82	23-82	49.0	45.6	9.9	9.7	49.0	46.0	44.6
Mexico	<40	153	102	73.2	47.2	59.3	18-39	25-39	30.3	32.4	5.5	4.4	31.0	33.0	
	≥40	56	114	26.8	52.8	40.7	40-78	40-65	49.3	48.0	7.6	6.1	48.0	48.0	
	Total	209	216	100.0	100.0	100.0	18-78	25-65	35.4	40.6	10.4	9.5	33.0	40.0	26.7
Poland	<40	174	152	82.5	75.2	45.1	19-39	19-39	29.3	29.2	5.1	5.3	28.5	29.0	
	≥40	37	50	17.5	24.8	54.9	40-62	40-66	46.2	47.4	5.8	5.8	45.0	46.0	
	Total	211	202	100.0	100.0	100.0	19-62	19-66	32.3	33.7	8.3	9.5	30.0	31.5	38.5
Russian Federation	<40	171	173	80.7	82.0	44.2	18-39	20-39	29.2	30.1	5.2	4.7	29.0	30.0	
	≥40	41	38	19.3	18.0	55.8	40-60	40-57	47.0	46.0	5.1	5.2	48.0	45.5	
	Total	212	211	100.0	100.0	100.0	18-60	20-57	32.6	33.0	8.7	7.8	30.0	31.0	38.5

Appendix A (continued). Age distributions of 22 samples across 11 countries.

Sample	Sub-sample	*N*		% of sample		% of nat'l pop.	Age range		Sample age *M*		Sample age *SD*		Sample age median		Nat'l age median
		wave 1	wave 2	wave 1	wave 2		wave 1	wave 2	wave 1	wave 2	wave 1	wave 2	wave 1	wave 2	
Singapore	<40	120	118	57.1	53.6	43.4	22-39	22-39	32.6	31.1	4.3	4.3	32.5	31.0	
	≥40	90	102	42.9	46.4	56.7	40-63	40-64	46.6	47.7	5.8	5.9	45.0	47.0	
	Total	210	220	100.0	100.0	100.0	22-63	22-64	38.6	38.8	8.6	9.7	38.0	38.0	40.1
Switzerland	<40	105	76	49.8	37.3	39.4	18-39	18-39	31.2	32.7	5.4	5.2	31.0	33.0	
	≥40	106	128	50.2	62.7	60.6	40-71	40-73	51.2	51.4	7.6	7.7	50.0	50.0	
	Total	211	204	100.0	100.0	100.0	18-71	18-73	41.3	44.5	12.0	11.3	40.0	45.0	41.7
U.K.	<40	65	68	30.5	30.4	40.9	20-39	21-39	32.6	31.1	4.4	4.8	33.0	31.0	
	≥40	148	156	69.5	69.6	59.1	40-80	40-82	52.1	54.4	8.6	11.1	52.0	53.0	
	Total	213	224	100.0	100.0	100.0	20-80	21-82	46.1	47.4	11.8	14.4	45.0	45.0	40.5
U.S.A.	<40	54	48	25.6	22.7	43.6	24-39	19-39	32.9	30.9	4.1	4.9	33.5	32.0	
	≥40	157	163	74.4	77.3	56.4	40-75	40-84	52.1	56.3	7.4	10.4	51.0	56.0	
	Total	211	211	100.0	100.0	100.0	24-75	19-84	47.2	50.5	10.7	14.2	48.0	52.0	36.8

Data were collected from 22 independent samples in 11 countries. Wave 1 was collected in 2010, wave 2 was collected in 2011.
N = sample size for given group; *M* = mean; *SD* = standard deviation; % of sample: proportion of group in country sample (within wave); % of nat'l pop. = proportion this group represents in the general population for that country; Nat'l age median = median age for the respective group within the general population of that country (from Central Intelligence Agency, 2008).
